# Risk Factors on Hypertensive Disorders among Jordanian Pregnant Women

**DOI:** 10.5539/gjhs.v6n2p138

**Published:** 2013-12-20

**Authors:** Amal K. Suleiman

**Affiliations:** 1 Pharmacy School, Department of Clinical Pharmacy, Princess Nora bint Abdul Rahman University, Kingdom of Saudi Arabia

**Keywords:** hypertension, pregnancy, risk factors, Jordan, Amman

## Abstract

Eight percent of pregnancies involve hypertensive disorders, which can have serious complications for mothers and children. There has only been minimal research into hypertension in pregnancy in developing countries, including Jordan. Therefore, this study aimed to identify how frequent certain risk factors that apply to hypertensive disorders during pregnancy were among women in the Jordanian capital of Amman. A prospective case-control study was conducted on 184 Jordanian pregnant patients with hypertensive disorders and 172 age-matched control subjects recruited from the maternity ward of a tertiary public hospital in Ammn city; they were followed-up until 85 days after the birth (late puerperium). A standardized questionnaire pilot-tested was completed by participants that included demographic data and known risk factors for hypertension in pregnancy. Statistical analysis SPSS was conducted to compare the frequency of risk factors using Fisher’s exact test, chi-square, Student’s t-tests, as well as multivariate logistic regression was conducted to identify independent risk factors. The results showed that chronic hypertension, prenatal hypertension, family history of preeclampsia, diabetes, high BMI, nulliparity, previous preeclampsia history and low education level were identified as risk factors for hypertensive disorders in pregnancy in this population; Moreover, diabetes, chronic hypertension and family history of preeclampsia were found to be independent risk factors. The results of the study contribute to the currently limited knowledge about the modifiable risk factors for hypertensive disorders during pregnancy among the Jordanian population, and could therefore be extremely useful for clinicians providing prenatal care.

## 1. Introduction

The most common pregnancy-associated disorder is hypertension, complicating 2-3% of pregnancies (Michael et al., 2012), occurs in 6–8 percent of all gestations (O’Brien et al., 2007; Helewa et al., 1997), it is a serious cause of maternal and mortality in developing countries where related as the most common medical problem in gestations (Mosca et al., 2011). In fact, not only developing countries are facing this medical problem, it has been found the most common medical problem in among developed countries women pregnancy. A study done by Colin (2012) in UK, found hypertensive disorders complicating up to 15% of pregnancies and a quarter of all antenatal admissions. Another study done by Chang et al. (2003) in the United States found that pregnancy-induced hypertension the major reason for 15.7% of maternal deaths, where Hedderson and Ferrara (2008) went further and fund can be a major risk of gestational diabetes mellitus.

In fact, hypertensive pregnancy disorders very seriously have been linked to increased perinatal and maternal morbidity and mortality. A study done by Dekker & Sibai (2001) where data obtained from the Nationwide Inpatient Sample of the Healthcare Cost and Utilization Project and the National Hospital Discharge Survey shows marked increases in the incidence of gestational hypertension and preeclampsia over the past two decades, and that an increasing number of women are entering pregnancy with chronic (preexisting) hypertension and they found also such cases have a markedly increased risk of severe adverse outcomes, including placental abruption and maternal cerebrovascular accidents, compared to normotensive women. It is therefore important to monitor blood pressure (BP) in pregnant women, in order to diagnosis and manage hypertensive disorders on time. Women with chronic hypertension (essential or secondary) need to be monitored frequently during pregnancy by an experienced obstetrician and physician. High BP in pregnancy may be a sign of preeclampsia, which is a serious condition that occurs in the second half of pregnancy and puerperium (O’Brien et al., 2007). The frequency rate of preeclampsia is 5% of pregnancies, and it is responsible for 16% of maternal deaths globally (WHO, 2003). Perinatal mortality doubles in women with preeclampsia (O’Brien et al., 2007). Preeclampsia is usually asymptomatic; it is detected by routine screening, and increased blood pressure and the presence of protein in the urine are considered to be indicators of preeclampsia (Wrobel et al., 2011)

Preeclampsia can range from mild to severe forms. In most cases, women progress slowly from one end of the spectrum to the other, and in some cases the disorder does not exceed mild preeclampsia. Progression in the most serious cases can be fulminant, with preeclampsia or eclampsia evolving from mild to severe within days, or even hours. Therefore, for the purposes of clinical management, it is important to over-diagnose preeclampsia, because the prevention of maternal and perinatal morbidity and mortality is a main goal, primarily through timing of delivery.

Hypertension during pregnancy, specifically pre-eclampsia, is an important obstetrical problem in less-developed countries (Amal & Syed, 2010; Dekker & Sibai, 2001). In particular, hypertensive disorders and their complications are the most common cause of maternal death in Middle East (WHO, 2003). Early diagnosis and treatment of this problem is important in pregnant women. However, many of the biophysical and biochemical tests that are currently used to identify women at risk are invasive, whereas others require expensive techniques or special expertise that precludes their utility in routine screening, and they generally have low levels of sensitivity and poor predictive values (Dekker & Sibai, 2001).

Another setback is that the risk factors for these hypertensive disorders are not clear in less developed countries; therefore, more research is required in this area (Amal & Syed, 2010; Dalmáz1 et al., 2011). The following factors have been reported for other populations: family history of preeclampsia and preeclampsia in a previous pregnancy (Caritis et al., 1998), chronic hypertension (Lykke et al., 2009), extremely young and old maternal age (Dekker & Sibai, 2001), obesity (O’Brien et al., 2007), Nulliparity and diabetes (Pipkin, 2001), and multifetal gestation (Wen et al., 2004).

If the risk factors in a population are known, patients predisposed to developing hypertensive disorders can be identified and subsequently administered adequate prenatal care (Amal & Syed, 2010). In this study, we have tried to better understand and identify the frequency of known risk factors for hypertensive disorders in pregnancy among Jordanian women, since the risk factors are not known for this population (Dalmáz1 et al., 2011). To the best of our knowledge, this is the first such study in the Jordanian population.

## 2. Method

This prospective case-control study was conducted on 184 Jordanian pregnant patients with hypertensive disorders and 172 age-matched control subjects. From June to August 2011, we recruited subjects from the maternity ward of a tertiary public hospital in the capital of Jordan; they were followed-up until 85 days after the birth (late puerperium), as preeclampsia can occur in the post-puerperal period. Hypertensive disorders were classified as for pregnancy based on American Guideline. These included mild or severe preeclampsia, non-proteinuric gestational hypertension, mild preeclampsia superimposed on chronic hypertension, and severe preeclampsia superimposed on chronic hypertension. A standardized questionnairewere pilot-tested was completed by participants that included demographic data and known risk factors for hypertension in pregnancy. The following risk factors were assessed: prenatal hypertension, smoking, low education level, chronic hypertension, diabetes, nulliparity, high BMI, multifetal gestation, previous preeclampsia history, and family history of preeclampsia. BMI were calculated from weight and height measurements obtained at the booking appointment. All samples had signed a written consent prior to their inclusion, and approval for the study protocol was obtained from the ethics committee of the hospital.

SPSS version 15.0 for Windows (SPSS Inc., Chicago, IL) was used for the statistical analyses. The Fisher’s exact test, chi-square test, and Student’s *t*-tests were used to compare the frequencies of risk factors between study groups depending on whether they were non-parametric or parametric variables. The variables tested in the univariate analysis included family and previous history of preeclampsia, multifetal gestation, BMI, nulliparity, diabetes, chronic hypertension, smoking habits and level of education. The continuous variable (BMI) was entered as a linear factor after being tested for nonlinearity. Multivariate logistic regression analysis was performed with a backward logistic regression analysis method to assess the independent roles of clinical, social and demographic variables that had been identified to be associated with hypertensive disease in pregnancy according to the univariate analysis. *P*-values<0.05 were considered to show statistical significance.

## 3. Results

This study raveled that were predominantly Jordanian (98%) with a mean age of 32 years (range, 22–43 years). The incidence of hypertensive disorders in pregnancy was as follows: mild preeclampsia, 51 (27.7%); severe preeclampsia, 48 (26.1%); eclampsia, 4 (2.1%); gestational hypertension, 8 (4.5%); and preeclampsia superimposed on chronic hypertension, 39.6%. As shown in Table 1 the demographical, clinical and social risk factors for hypertensive disorders. Family history of preeclampsia (PE), previous PE history, high BMI, diabetes, chronic hypertension, low level of education and prenatal hypertension were found in a higher number of women with hypertensive disorders in pregnancy than normotensive women. Nulliparity, multifetal gestation and smoking habits were not significantly different between the patients and controls, although multifetal gestation showed a higher frequency in the patient group.

**Table 1:** Risk factors for hypertensive disorders in pregnancy

Characteristic	Hypertensive disorder in pregnancy n = 184	Control n = 172	p
Prenatal hypertension	91%	95%	0.005
Smoking	16%	25%	0.381
Low education level	33%	54%	0.004
Chronic hypertension	34.5%	7%	0.002
Diabetes	24%	9%	0.002
Nulliparity	29%	25%	0.403
High BMI (kg/m²)*	34.8 ± 5.2	25.4 ± 3.6	0.001
Multifetal gestation	8%	4%	0.511
Previous PE history	53%	5%	0.000
Family history of PE	41%	17%	0.002

PE, preeclampsia; BMI, body mass index;

* Data are presented as mean ± SD or %.

The results of the univariate logistic regression analysis for characteristics of women with hypertensive disorders in pregnancy compared to normotensive women are shown in Table 2. Diabetes, nulliparity, family history of PE, chronic hypertension, low educational level, prenatal hypertension and high BMI were significantly associated with hypertensive diseases in pregnancy. Of note, smoking and multifetal gestations were not significantly associated. The multivariate analysis found that chronic hypertension, diabetes and a family history of PE remained significantly associated with gestational hypertension (Table 3).

**Table 2:** Univariate analysis - women with hypertensive disorders in pregnancy characteristics compared to normotensive women

Characteristic	Odds ratio (95% CI)	P
Prenatal hypertension	0.23 (0.07–0.69)	0.015
Smoking	0.69 (0.39–1.33)	0.699
Low education level	0.40 (0.18–0.69)	0.001
Chronic hypertension	9.11 (4.00–20.02)	0.001
Diabetes	5.01 (2.00–11.09)	0.001
Nulliparity	1.98 (1.22–4.08)	0.019
High BMI (kg/m²)*	2.00 (1.14–1.33)	0.002
Multifetal gestation	1.55 (0.49–4.98)	0.387
Previous PE history	18.03 (8.02–39.49)	0.001
Family history of PE	4.02 (1.83–6.19)	0.001

(**p<0.01, *p<0.05)

**Table 3:** Multivariate analysis - women with hypertensive disorders in pregnancy characteristics compared to normotensive women

Characteristic	Odds ratio (95% CI)	p
Diabetes	4.11 (1.84–13.02)	0.019
Family history of PE	4.00 (1.62–9.11)	0.001
Chronic hypertension	6.98 (2.07–25.08)	0.002

(**p<0.01, *p<0.05)

## 4. Discussion

As for our result in this study, we found that both variables multifetal gestation and smoking habits had no significant relation on PE; in time, these factors have been described as risk factors by some reports (Dalmáz1 et al., 2011). For example, Pipkin (2001) reported that multiple pregnancies doubled the risk of PE. Another curious but consistent finding reported by Cooper et al. (1988) is that women who smoke in less level of risk in developing PE than women who do not smoke. However, this benefit was negated by the substantial negative impact of smoking on growth of the fetus, and general health. In our population, this “protective” effect was not observed. It is possible that we did not obtain similar results because of the small sample size. In addition, extreme maternal age was not a risk factor as per our results because the participants were age-matched; nevertheless, it is an established risk factor for PE (Dalmáz1 et al., 2011). To study maternal age as a risk factor for this condition, in the future can be considered and further investigates the factors have not been associated such as the smoking.

Furthermore, we found chronic hypertension, family history of PE, diabetes, high BMI, nulliparity, previous history of PE and low education level to be significantly associated with hypertensive disease in pregnancy among Jordanian women. The risk factors identified here are similar to those reported by other studies conducted in different populations (Sibai et al., 2005; Pipkin, 2001; Lykke et al., 2009; Hutcheon et al., 2011; Dalmáz1 et al., 2011). In particular, we found a family history of PE, diabetes and chronic hypertension to be independent risk factors. This has also been reported in other studies (Araujo et al., 2007; Bezerra et al., 2010; Dalmáz1 et al., 2011).

Similar to our findings, involvement of the genetic component in pathophysiological abnormalities associated with PE has been suggested before (Mütze et al., 2008). For example, PE was more common in women whose mothers had experienced PE during pregnancy (Cooper et al., 1988) and in pregnancies fathered by sons of women who had PE (Assis et al., 2008); from these findings, it seems that both maternal and fetal genes play a role in this syndrome. Therefore, pregnant women with a family history of PE it should monitored carefullyboth prenatally and in the postpartum period (Dalmáz1 et al., 2011).

With regard to the influence of BMI, O’Brien et al. (2007) also reported that the risk of PE increases with increase in BMI. The reason for this is probably the increased shear stress caused by hyperdynamic circulation which is associated with obesity (Sibai et al., 2005). Dalmáz et al. (2011) also mentioned that being overweight (obesity and pre-obesity) was correlatedwith an increased risk of developing PE. In addition, a study conducted by Gaio et al. (2001) found obesity to be a risk factor for PE/eclampsia and chronic hypertension, and Assis et al. (2008) demonstrated that obesity increases the risk of gestational hypertension and PE superimposed on chronic hypertension. The worldwide increase in the frequency of obesity is likely to also increase the frequency of PE. Therefore, public health measures should be taken to prevent and/or treat obesity as it could consequently prevent hypertensive disorders and their related illnesses. With regard to diabetes as a risk factor (Schmidt et al., 2001), where a study conducted by Monira et al. (2010) in Palestine have found that gestational DM is independently correlated. Moreover, preexisting diabetes mellitus has also been reported as a risk factor (Pipkin, 2001). As stated before, women with preexisting chronic hypertension also have an increased risk of PE (Bezerra et al., 2010; Lykke et al., 2009; Audibert, et al., 2010; Assis et al., 2008). Thus, public health departments should focus on the prevention and treatment of these diseases and consequently prevent the occurrence of related diseases such as PE.

In general, PE occurs during the first pregnancy, with frequency rate of 2–7% in healthy nulliparous women (Haelterman et al., 2003). Nulliparity in pregnancyis well known as a risk factor for hypertensive disorders (Sibai et al., 2005). In this study, we also determined that nulliparity was an independent risk factor according to the logistic regression analysis, whereby nulliparous women had a two-fold increased risk of developing a hypertensive disorder during their pregnancy. In our study, we found that a significantly higher number of hypertensive women had a low educational level. In agreement with this, Haelterman et al. (2003) showed that PE has a higher frequency in socially disadvantaged women; therefore, health services should be more attentive to the specific needs of these women. In our study, we found prenatal hypertension, low education level, chronic hypertension, diabetes, high BMI, previous PE history, and family history of PE factors associated with prenatal care to influence the development of hypertensive disorders in pregnancy; therefore, the importance and the protective effect of prenatal care cannot be denied. In the prenatal care the following factors are analyzed, among others: schooling, familiar and previous history of hypertension and diabetes, number of pregnancies, and smoking. Some of these factors were demonstrated to influence the development of hypertensive disorders in pregnancy. Women who receive adequate help can be aware of these possible risk factors earlier and can undertake preventive actions, therefore decreasing the chances of developing such diseases. Our results indicate that women who do not receive prenatal care have a four-fold increased risk of developing hypertensive disorders in pregnancy. Therefore, it is important for public health initiatives to aim at improving prenatal access, especially among socially disadvantaged women with low education levels.

## 5. Conclusion

The results of the present research showed and revealed that family and a previous history of PE, high BMI, diabetes and chronic hypertension are risk factors for hypertensive disorders in pregnancy among Jordanian women. Our improved knowledge of important risk factors in the Jordanian population could help clinicians to identify pregnant women who are at risk of PE. In pregnancy, the prevention of hypertensive diseases will markedly improve antenatal careperhaps have a great potentialfor these diseasestreatment of. Primary care should reach all pregnant women to educate and aware of the Risk factors for hypertensive disorders in pregnancy. In Jordan, still there is a short inrecognition about how to reach all pregnantwomen with needed preventive interventions. Action is important for the implementation of programs that pending the issue related to hypertensive disorder in Jordanian pregnant women.
